# Asymmetrical methyltransferase PRMT3 regulates human mesenchymal stem cell osteogenesis via miR-3648

**DOI:** 10.1038/s41419-019-1815-7

**Published:** 2019-08-05

**Authors:** Zhang Min, Liu Xiaomeng, Li Zheng, Du Yangge, Liu Xuejiao, Lv Longwei, Zhang Xiao, Liu Yunsong, Zhang Ping, Zhou Yongsheng

**Affiliations:** 1 Department of Prosthodontics, Peking University School and Hospital of Stomatology, 100081 Beijing, China; 2 National Engineering Lab for Digital and Material Technology of Stomatology, National Clinical Research Center for Oral Diseases, Peking University School and Hospital of Stomatology, 100081 Beijing, China; 3 Beijing Advanced Innovation center for Genomics (ICG), College of Life Sciences, Peking University, 100871 Beijing, China

**Keywords:** Mesenchymal stem cells, Stem-cell differentiation

## Abstract

Histone arginine methylation, which is catalyzed by protein arginine methyltransferases (PRMTs), plays a key regulatory role in various biological processes. Several PRMTs are involved in skeletal development; however, their role in the osteogenic differentiation of mesenchymal stem cells (MSCs) is not completely clear. In this study, we aimed to elucidate the function of PRMT3, a type-I PRMT that catalyzes the formation of ω-mono- or asymmetric dimethyl arginine, in MSCs osteogenesis. We found that PRMT3 promoted MSCs osteogenic commitment and bone remodeling. PRMT3 activated the expression of miR-3648 by enhancing histone H4 arginine 3 asymmetric dimethylation (H4R3me2a) levels at promoter region of the gene. Overexpression of miR-3648 rescued impaired osteogenesis in PRMT3-deficient cells. Moreover, administration of *Prmt3* shRNA or a chemical inhibitor of PRMT3 (SGC707) caused an osteopenia phenotype in mice. These results indicate that PRMT3 is a potential therapeutic target for the treatment of bone regeneration and osteopenia disorders.

## Introduction

Bone is a dynamic organ that undergoes persistent remodeling over the course of life. Bone homeostasis and remodeling mainly involves bone destruction executed by osteoclasts, and bone deposition mediated by osteoblasts derived from mesenchymal stem cells (MSCs)^1,2^. This complex process requires balance and synchronized regulation at different control levels. MSCs are multipotent cells with the ability to develop into various cell types, such as osteoblasts, adipocytes, and chondrocytes under defined conditions^3^. Diminished MSC differentiation contributes to the etiology of osteopenic diseases associated with aging, post-menopause, and diabetes mellitus^4–6^. MSCs are a promising prospect for regenerative medicine, especially for bone repair^7–9^. The mechanism underlying osteogenesis, however, remains largely unknown. This is an impediment to the clinical application of MSCs. Understanding the molecular mechanisms of MSC lineage commitment is therefore essential for developing novel therapies for diseases involving reduced MSCs function.

Arginine methylation is a widespread post-translational modification, and is as common as ubiquitination and phosphorylation. Protein methylation mediated by protein arginine methyltransferases (PRMTs) influences various biological processes, including RNA and protein subcellular localization, transduction of signals, RNA splicing, transcription and chromatin remodeling, and DNA repair. PRMTs, act on a wide range of substrates, including histones and non-histones^10–13^. PRMTs play important roles in cell fate determination of blood progenitors and diverse metabolic processes associated with human diseases^14–18^. PRMTs play critical roles in skeletal development and bone homeostasis. PRMT1 modulates craniofacial bone formation as an upstream regulator of Msx1^19^. PRMT1 is an indispensable regulator of RANKL-induced osteoclast differentiation. PRMT1 activated osteoclast function and led to bone loss in OVX mice^20^. PRMT5 activates RANKL-induced osteoclast differentiation, which involves the regulation of CXCL10 and RSAD2^21^. Pharmacological inhibition of Prmt5 in the ST-2 and W-20 cell lines inhibited colony-forming units and promoted osteogenesis^22^. Thus, arginine methylation is essential for the tight regulation of cell fate decision during embryonic development and homeostasis maintenance.

PRMT3 is a type-I PRMT that catalyzes the formation of ω-mono- or asymmetric dimethyl arginine, and has a unique substrate-binding C_2_H_2_ Zn finger domain at its N-terminus and a catalytic domain at its C-terminus^23,24^. RpS2 was first reported as the substrate of PRMT3 in the cytoplasm^25,26^. *Prmt3* knock-out cell lines exhibited decreased rpS2 methylation levels^27^. PRMT3 also modulates dendritic spine maturation in rats by interaction with rpS2^28^. PRMT3 was related to hepatic lipogenesis as a cofactor to LXRα in the nucleus. High expression levels of PRMT3 are associated with non-alcoholic fatty liver disease (NAFLD)^29^. PRMT3 methylates PABPN1 and is involved in oculopharyngeal muscular dystrophy, resulting from polyalanine expansion in PABPN1^30,31^. DAL-1/4.1B, a tumor suppressor, interacts with PRMT3 and suppresses its methyltransferase activity, indicating a potential role of PRMT3 in control of tumor growth^32,33^. In patients with atherosclerosis, PRMT3 expression was increased in myocardial tissues, along with downregulation of genes that promotes NO synthesis^34^. However, the role of PRMT3 in skeletal development has not been studied. *Prmt3*–null mice exhibited delayed embryonic development; the embryos were significantly smaller than those of wild-type mice, but could survive and achieve normal size during adulthood^27^. We aimed to investigate the potential effects of PRMT3 on osteoblastic commitment of MSCs and maintenance of bone homeostasis.

In the current study, we observed that PRMT3 is an essential regulator of MSC-mediated osteogenesis and bone homeostasis. Importantly, PRMT3 promoted the osteogenic differentiation of MSCs by increasing H4R3me2a levels in the DNA regions 343 bp upstream and 505 bp downstream of the transcription starting site (TSS) of miR-3648, which were likely promoter regions. PRMT3 deficiency contributed to bone loss in mice. Our findings uncovered a potential mechanism by which PRMT3 affects osteogenic differentiation of MSCs, and suggested that targeting PRMT3 might be an effective therapeutic strategy to treat bone metabolic disease and in bone regenerative medicine.

## Results

### PRMT3 and H4R3me2a could be involved in MSCs osteogenesis

**Fig. 1:**
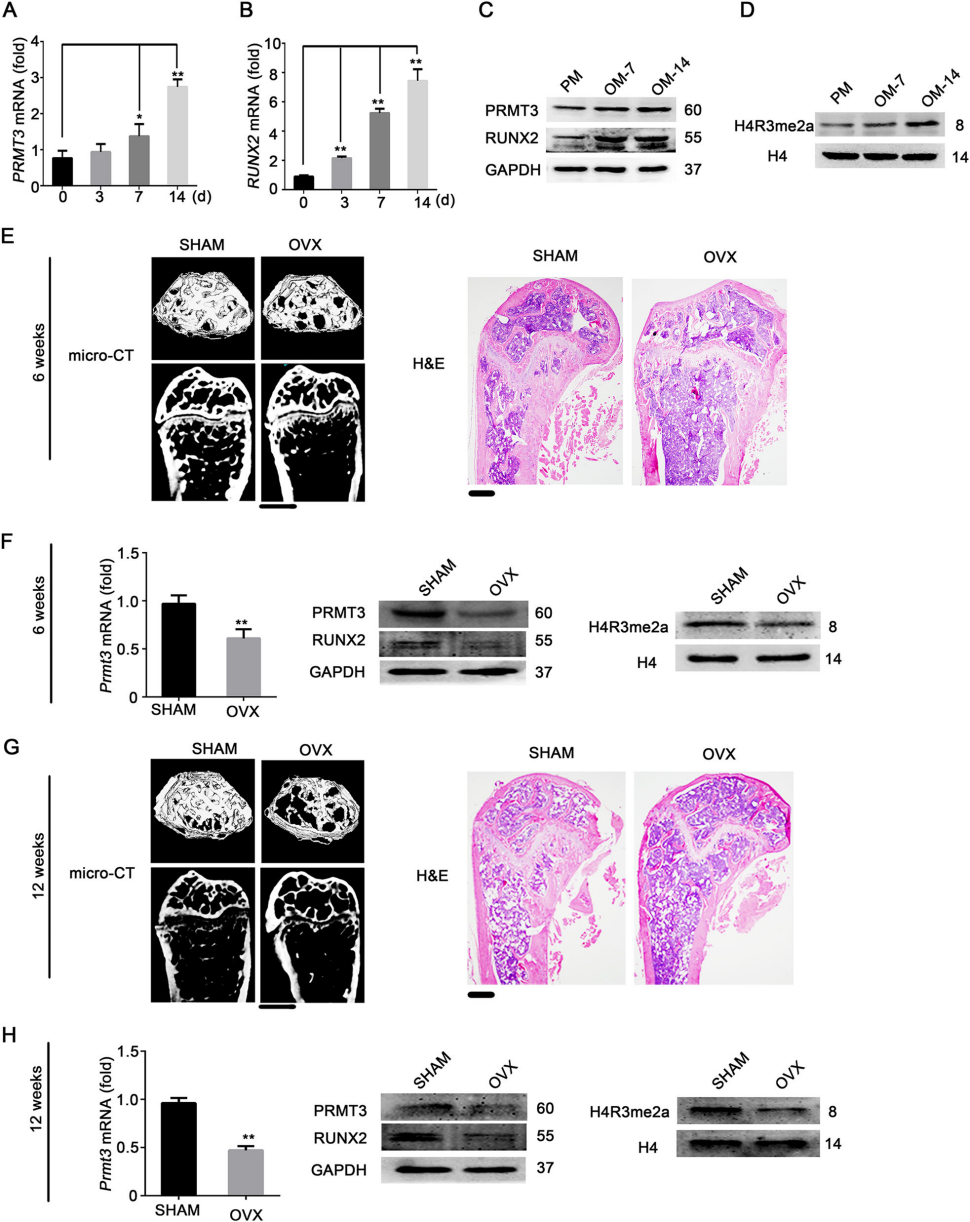
PRMT3 and H4R3me2a are likely involved in osteogenesis. **a**, **b** Expression profiles of *PRMT3* (**a**) and *RUNX2* (**b**) during hMSCs osteogenic differentiation, determined by qRT-PCR analysis. Data are shown as mean ± SD; *n* = 3 independent experiments; *: compared with day 0, *P* < 0.05; **: compared with day 0, *P* < 0.01 by Student’s *t*-tests. **c** Western blotting analysis of PRMT3 and RUNX2 protein levels during osteoblastic differentiation of hMSCs. GAPDH was used as an internal control. PM, proliferation medium; OM, osteogenic medium. **d** Western blotting analysis of dimethyl-asymmetric methylation level of H4R3 during osteogenic differentiation of hMSCs. H4 was used as an internal control. **e** Micro-CT image and H&E staining of OVX mice at 6 weeks. Scale bar for micro-CT image: 1 mm; scale bar for H&E staining: 200 μm. **f** qRT-PCR analysis of *Prmt3* mRNA level (left), western blotting analysis of PRMT3 and RUNX2 protein levels (middle), and H4R3me2a (right) in BMMSCs of OVX mice at 6 weeks. Data are shown as mean ± SD; *n* = 3 independent experiments; **P < 0.01 compared with SHAM group by Student’s *t*-tests. **g** Micro-CT image and H&E staining of OVX mice at 12 weeks. Scale bar for micro-CT image: 1 mm; scale bar for H&E staining: 200 μm. **h** qRT-PCR analysis of *Prmt3* mRNA level (left), western blotting analysis of PRMT3 and RUNX2 protein levels (middle), and H4R3me2a (right) in BMMSCs of OVX mice at 12 weeks. Data are shown as mean ± SD; *n* = 3 independent experiments; **P < 0.01 compared with SHAM group by Student’s *t*-tests

To investigate the potential role of PRMT3 in osteoblastic commitment of MSCs, the expression of PRMT3 in human MSCs was detected after osteoinductive culture. As shown in Fig. 1a–c, qRT-PCR and western blotting analysis revealed that PRMT3 was strongly induced, along with the upregulation of the osteogenic marker RUNX2. In addition, we found that histone H4 dimethyl Arg3 asymmetric methylation (H4R3me2a) catalyzed by PRMT3 was upregulated after osteogenic induction (Fig. 1d). To assess the potential role of PRMT3 in bone homeostasis, we created ovariectomized (OVX) model mice. We then determined whether the expression level of PRMT3 was altered in impaired MSCs. Microcomputed tomography (micro-CT) analysis and hematoxylin and eosin (H&E) staining exhibited that the trabecular area in femurs were significantly reduced in the OVX group compared to those in the SHAM group at 6 (Fig. 1e and Supplementary Fig. 1a) and 12 weeks after operation (Fig. 1g and Supplementary Fig. 1b). Next, BMMSCs were isolated from mouse bone marrow by the traditional flush method. As shown in Supplementary Fig. 1c, d, BMMSCs from OVX mice showed impaired osteogenic ability based on ALP staining and quantification. We then checked the expression of PRMT3 and H4R3me2a in BMMSCs derived from OVX mice. As shown in Fig. 1f, h, PRMT3 and H4R3me2a expression levels were significantly decreased in BMMSCs from OVX mice, which were consistently correlated with the RUNX2 levels.

### PRMT3 arginine methyltransferase activity is required for the promotion of osteoblastic differentiation of MSCs

**Fig. 2:**
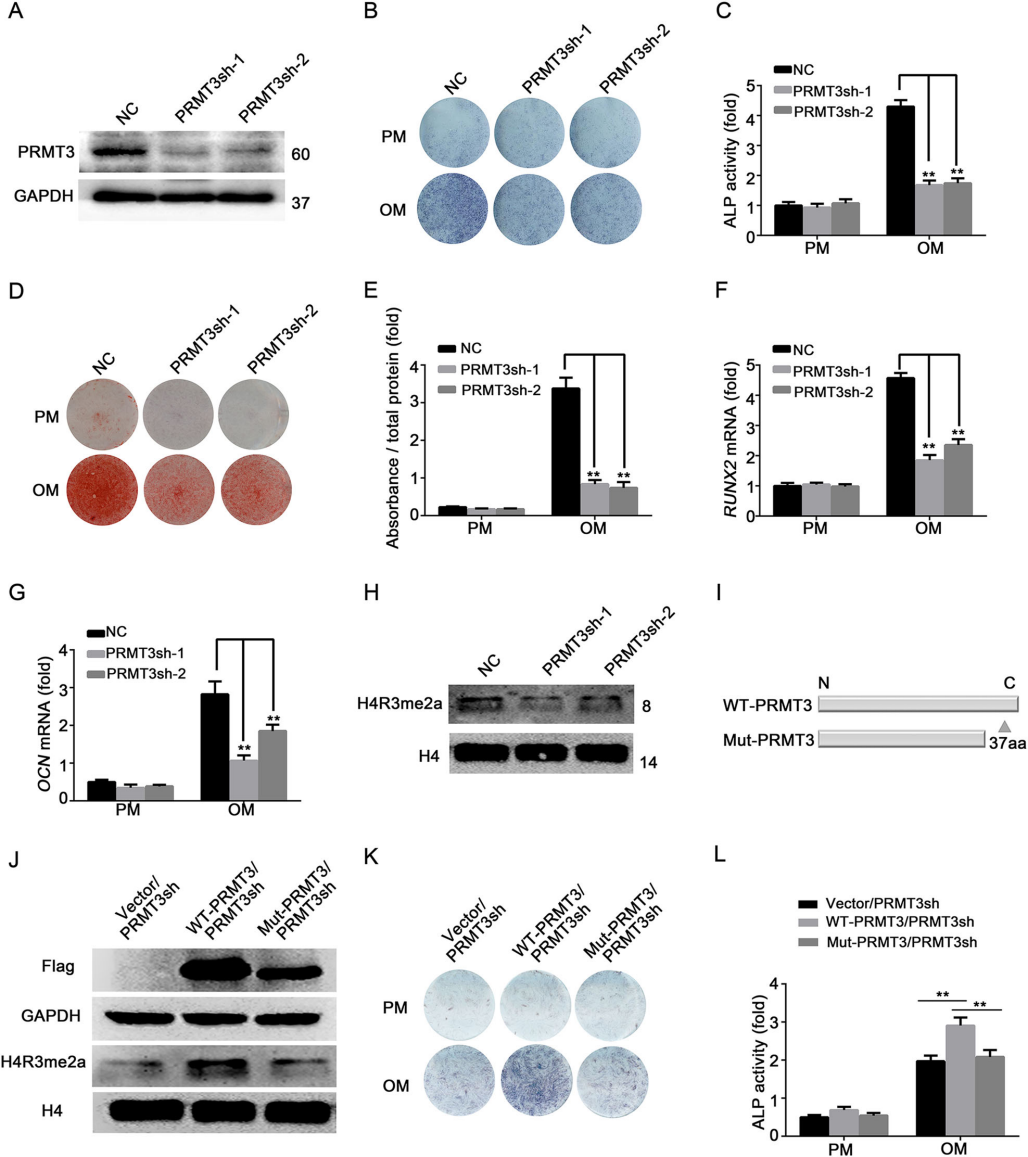
PRMT3 arginine methyltransferase activity is essential for the PRMT3-mediated promotion of MSCs osteogenic differentiation. **a** Western blotting analysis of PRMT3 knockdown efficiency; GAPDH was used as internal control. **b**, **c** ALP activity was significantly suppressed by knockdown of PRMT3 at 7 days after osteogenic induction, as indicated by ALP staining (**b**) and quantification (**c**). Data are shown as mean ± SD; *n* = 3 independent experiments; **P < 0.01 compared with NC-OM by Student’s *t*-tests. **d**, **e** PRMT3 knockdown exhibited decreased extracellular matrix mineralization in cells at 14 days after osteogenic induction, as shown by Alizarin Red S staining (**d**) and calcium quantitative analysis (**e**). Data are shown as mean ± SD; *n* = 3 independent experiments; **P < 0.01 compared with NC-OM by Student’s *t*-tests. **f**, **g** Knockdown of PRMT3 inhibited the expressions of *RUNX2* (**f**) and *OCN* (**g**) as determined by qRT-PCR. Data are shown as mean ± SD; *n* = 3 independent experiments; **P < 0.01 compared with NC-OM by Student’s *t*-tests. **h** Western blotting analysis of H4R3me2a expression level in PRMT3 knockdown cells. H4 was used as an internal control. **i** Schematic illustration of catalytic dead mutant PRMT3. **j** Western blotting analysis of H4R3me2a expression level with forced expression of wild-type or mutant PRMT3 in PRMT3sh cells. H4 was used as an internal control. **k**, **l** ALP staining (**k**) and quantification (**l**) of PRMT3 rescue cell lines after 7-day osteogenic differentiation. Data are shown as mean ± SD; *n* = 3 independent experiments; **P < 0.01 by Student’s *t*-tests

To explore the role of PRMT3 in MSCs fate determination, we constructed stable *PRMT3* knockdown MSCs by using lentiviruses expressing *PRMT3* shRNA. Two shRNA sequences against *PRMT3* were designed to exclude off-target effects. The majority of infected cells were GFP-positive (Supplementary Fig. 2a). The knockdown efficiency of PRMT3 was evaluated by qRT-PCR (Supplementary Fig. 2b) and western blotting (Fig. 2a). After treating MSCs with osteoinductive media for 1 week, ALP activity was greatly suppressed by knockdown of PRMT3 (Fig. 2b, c). In addition, alizarin red S staining and quantification indicated reduced calcium deposition in PRMT3 knockdown cells 14 days after osteogenic induction (Fig. 2d, e). Furthermore, knockdown of PRMT3 decreased the expression levels of the osteoblastic differentiation indicators *RUNX2* and *OCN* (Fig. 2f, g). Subsequently, we determined whether the arginine methyltransferase activity of PRMT3 was involved in the modulation of osteoblastic commitment. The H4R3me2a expression levels were reduced in PRMT3 knockdown cells (Fig. 2h). In addition, a catalytically dead mutant of PRMT3 (deletion of 37 amino acid in C-terminal) was generated (Fig. 2i). We also constructed a PRMT3 rescue cell line by transfecting the vector, Flag-PRMT3 (wild-type, WT), and Flag-PRMT3 mutant (Mut) in MSCs/PRMT3sh (Supplementary Fig. 2c). Wild-type PRMT3 could rescue the expression of H4R3me2a in PRMT3sh cells, while mutant PRMT3 could not (Fig. 2j). Moreover, only wild-type PRMT3 but not mutant PRMT3 could promote osteoblastic differentiation, as shown by ALP staining and quantification (Fig. 2k, l). Thus, our data suggested that PRMT3 could promote the osteogenic differentiation of MSCs through its arginine methyltransferase activity.

### PRMT3 is a positive regulator of MSC-mediated bone formation in vivo

**Fig. 3:**
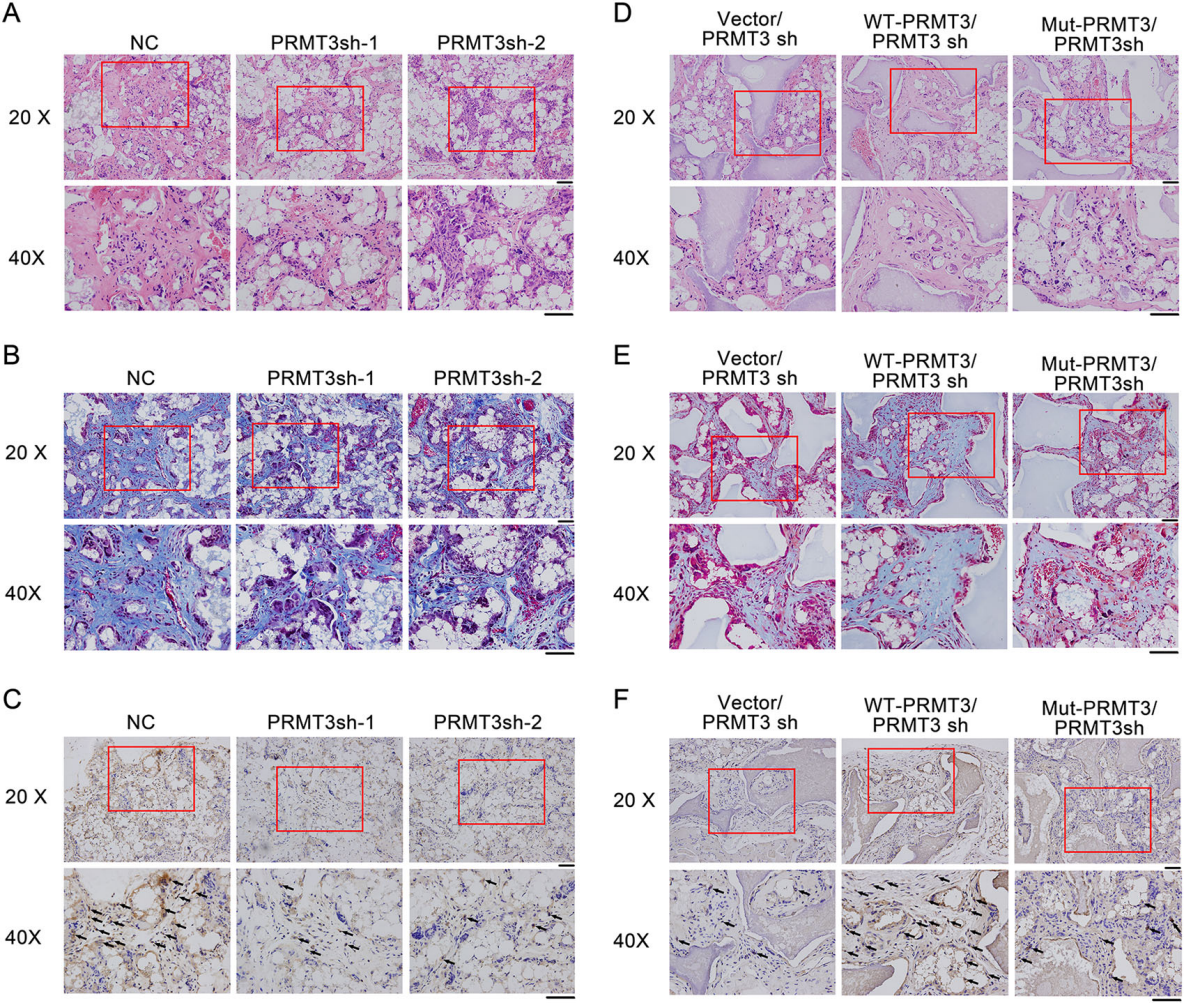
PRMT3 is a positive regulator of MSC-mediated bone formation in vivo. **a** H&E staining of PRMT3 knockdown cells. **b** Masson’s trichrome staining of PRMT3 knockdown cells. **c** IHC staining for OCN in PRMT3 knockdown cells. **d** H&E staining of PRMT3 rescue cells. **e** Masson trichrome staining of PRMT3 rescue cells. **f** IHC staining for OCN in PRMT3 rescue cells. H&E staining, hematoxylin-eosin staining; IHC staining, immunohistochemistry staining; Scale bars represents 50 μm; Black arrows indicate positive staining of OCN; *n* = 6

To confirm the effect of PRMT3 in the osteoblastic differentiation of MSCs, we mixed the MSCs/PRMT3sh with beta-tricalcium phosphate (β-TCP) particles, and implanted the hybrids into the subcutaneous tissue of nude mice. H&E staining indicated that deficiency of PRMT3 led to less bone-like tissue formation compared with the controls (Fig. 3a). Consistent with this, Masson’s trichrome staining displayed less collagen deposition (Fig. 3b). Immunohistochemical (IHC) staining indicated less positive staining for osteogenic marker OCN was found in the PRMT3sh group (Fig. 3c). Next, we performed the same experiments with the PRMT3 rescue cell line. As shown in Fig. 3d–f, only wild-type PRMT3 could elevate the osteogenic capacity of PRMT3sh cells in vivo, which corroborated the in vitro data. These data suggested that the arginine methyltransferase activity of PRMT3 was necessary for the modulation of osteoblastic differentiation.

### PRMT3 modulates the osteoblastic differentiation of MSCs by targeting miR-3648

**Fig. 4:**
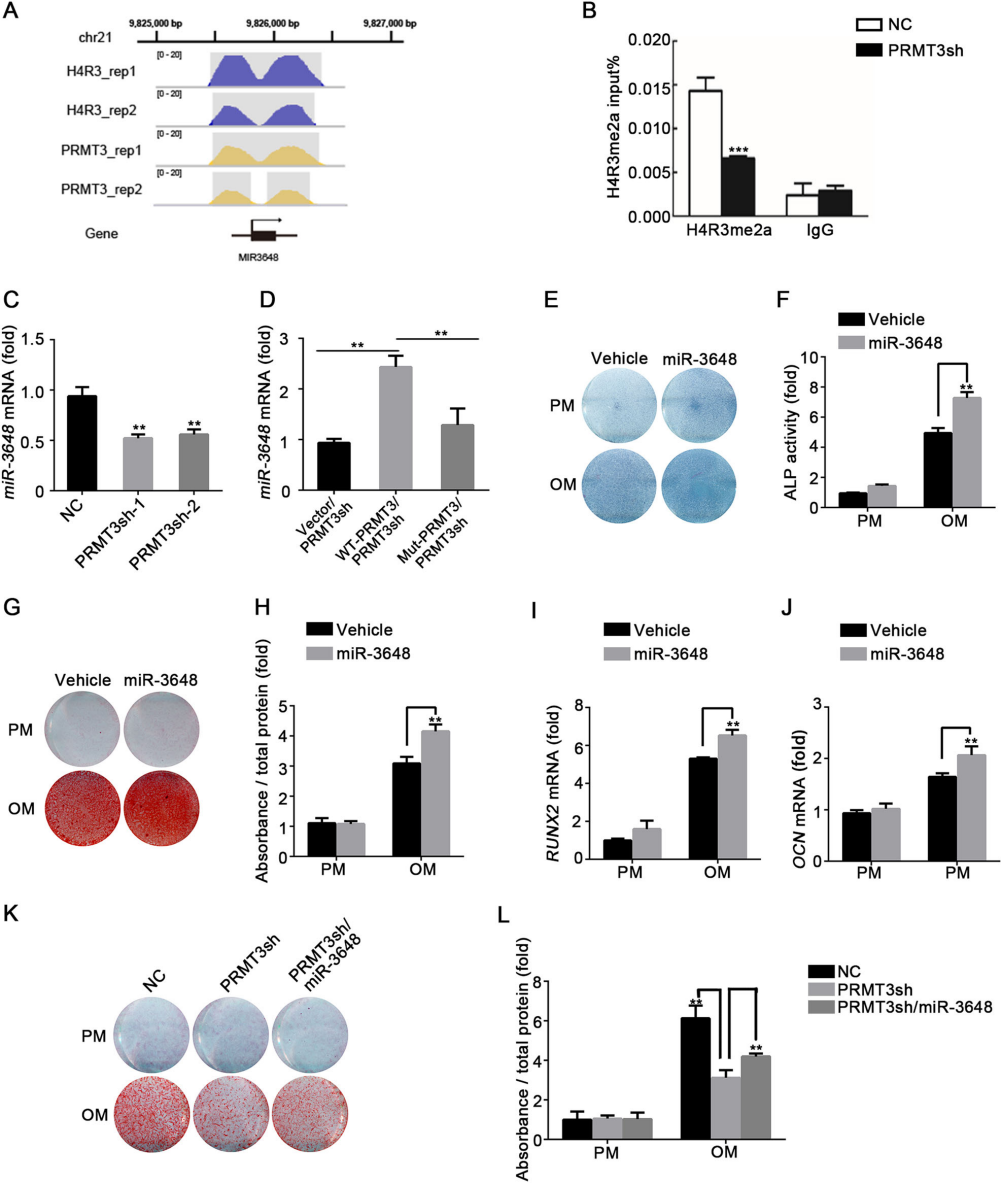
PRMT3 modulates MSCs osteogenic differentiation by targeting miR-3648. **a** H4R3me2a and PRMT3 signal tracks for the loci of miR-3648. The gray shaded part indicates H4R3me2a or PRMT3 enriched region obtained by peak calling. **b** ChIP analysis showed H4R3me2a at the promoter of miR-3648 in control and PRMT3 knockdown cells. Data are shown as mean ± SD; *n* = 3 independent experiments; ***P < 0.001 compared with NC by Student’s *t*-tests. **c** Relative expression level of miR-3648 in PRMT3 knockdown MSCs, as determined by qRT-PCR. Data are shown as mean ± SD; *n* = 3; **P < 0.01 compared with NC by Student’s *t*-tests. **d** qRT-PCR analysis of miR-3648 expression in PRMT3sh MSCs with forced expression of wild-type or mutant PRMT3. Data are shown as mean ± SD; *n* = 3; **P < 0.01 by Student’s *t*-tests. **e**, **f** ALP staining (**e**) and quantification (**f**) in miR-3648 overexpression cells on day 7 after osteogenic induction. Data are shown as mean ± SD; *n* = 3; **P < 0.01 by Student’s *t*-tests. **g**, **h** Alizarin Red S staining (**g**) and quantification analysis (**h**) in miR-3648 overexpression cells on day 14 after osteogenic induction. Data are shown as mean ± SD; *n* = 3; **P < 0.01 by Student’s *t*-tests. **i**, **j** Overexpression of miR-3648 promoted the expression of *RUNX2* (**i**) on day 7 and *OCN* (**j**) on day 14 after osteogenic induction, as determined by qRT-PCR. Data are shown as mean ± SD; *n* = 3; **P < 0.01 by Student’s *t*-tests. **k**, **l** ARS staining (**k**) and quantification (**l**) revealed that overexpression of miR-3648 elevated the mineralized nodule formation in PRMT3 knockdown cells on day 14 after osteogenic induction. Data are shown as mean ± SD, *n* = 3. **P < 0.01

In order to elucidate the key mechanisms underlying PRMT3-mediated modulation of MSCs osteogenesis, we performed ChIP-seq to determine the potential targets of PRMT3 and H4R3me2a. As shown in Table 1A, B, 17 PRMT3 and 29 H4R3me2a enriched regions were found, wherein miR-3648 was determined as the co-binding site of PRMT3 and H4R3me2a (Fig. 4a). ChIP-qPCR results showed that PRMT3 knockdown decreased H4R3me2a levels in the promoter region of miR-3648 (Fig. 4b). To confirm whether miR-3648 expression is directly regulated by PRMT3, we examined miR-3648 expression in PRMT3 knockdown cells. As shown in Fig. 4c, miR-3648 was significantly inhibited by PRMT3 deficiency. We also detected miR-3648 in the PRMT3 rescue cell line, and found that forced expression of wild-type but not mutant PRMT3 increased the expression level of miR-3648 (Fig. 4d). To explore the function of miR-3648 in osteogenic differentiation, we treated MSCs with miR-3648 mimic or inhibitor—the overexpression or knockdown efficiency is shown in Supplementary Fig. 3a–b. Overexpression of miR-3648 enhanced ALP activity (Fig. 4e, f) and calcium deposition (Fig. 4g, h) in MSCs treated with osteogenic inducer. The mRNA expression levels of *RUNX2* and *OCN* were also increased in the miR-3648 treated group (Fig. 4i, j). In addition, MSCs treated with miR-3648 inhibitor showed reduced ALP activity (Supplementary Fig. 3c–d) and calcium deposition (Supplementary Fig. 3e–f). Furthermore, we overexpressed miR-3648 in PRMT3 knockdown cells to analyze whether miR-3648 could rescue impaired osteogenic capacity. ARS staining and quantification revealed that overexpression of miR-3648 significantly elevated mineralized nodule formation in PRMT3 knockdown cells (Fig. 4k, l). Collectively, our data suggest that PRMT3 promotes osteogenesis by modulating the expression of miR-3648.

**Table 1A:** PRMT3 enriched regions obtained by peak calling

Chr	Start	End	Length	Abs summit	Pileup	Fold enrichment
chr1	91852810	91853102	293	91852969	106.94	26.98379
chr1	156186298	156186560	263	156186431	93.4	9.44046
chr10	42389330	42389534	205	42389480	117.41	2.19279
chr11	85195028	85195224	197	85195101	45.39	11.59822
chr12	49578773	49578862	90	49578799	10.91	8.81699
chr12	127650485	127650648	164	127650560	21.82	11.41175
chr16	33963053	33963318	266	33963121	44.52	9.10399
chr16	33963887	33964033	147	33963968	51.5	27.6334
chr17	17286832	17286967	136	17286914	14.84	9.99368
chr19	27732021	27732268	248	27732234	93.4	2.55148
chr2	133025749	133025850	102	133025826	24.88	8.62626
chr21	9825493	9826367	875	9826191	78.56	7.95646
chr21	9826842	9827550	709	9827369	117.41	7.89403
chr5	71146767	71146900	134	71146850	24.88	8.62626
chr8	70602325	70602492	168	70602382	57.18	24.75641
chrX	12995051	12995176	126	12995132	17.02	13.16459
chrX	108297370	108297815	446	108297726	63.72	32.36231

**Table 1B:** H4R3me2a enriched regions obtained by peak calling

Chr	Start	End	Length	Abs summit	Pileup	Fold enrichment
chr1	91852803	91853114	312	91852974	121.67	24.53474
chr1	97144579	97144683	105	97144638	16.94	8.96813
chr1	121483314	121483403	90	121483376	82.01	2.76691
chr1	121483783	121483880	98	121483817	79.33	2.86904
chr1	156186291	156186584	294	156186448	102.06	10.30633
chr10	98510372	98510509	138	98510420	22.28	15.11984
chr11	65266876	65267089	214	65266980	28.08	17.8396
chr11	82400759	82400853	95	82400799	13.37	9.97275
chr11	85195027	85195223	197	85195101	52.15	13.28647
chr12	49578714	49578962	249	49578796	22.73	17.56495
chr12	104659396	104659510	115	104659434	12.03	9.64742
chr12	118582745	118582867	123	118582817	11.59	8.68136
chr12	127650481	127650648	168	127650560	25.85	13.42504
chr16	33963047	33963323	277	33963122	57.49	11.69883
chr16	33963883	33964036	154	33963967	60.61	32.42842
chr17	17286811	17287010	200	17286900	24.07	15.81534
chr18	108194	108484	291	108238	109.19	2.68767
chr18	6462268	6462364	97	6462335	16.04	12.73486
chr19	27732017	27732275	259	27732047	98.94	3.8813
chr2	133025747	133025856	110	133025826	28.97	9.98997
chr21	9825481	9826366	886	9825624	94.93	9.59322
chr21	9826836	9827553	718	9827305	129.25	7.23614
chr5	71146756	71146894	139	71146830	29.42	15.20781
chr7	44507686	44507803	118	44507742	15.6	12.40187
chr7	132719500	132719624	125	132719536	10.25	5.62545
chr8	70602324	70602495	172	70602389	58.83	25.4601
chr9	79186754	79186872	119	79186805	10.25	8.40596
chrX	12994994	12995207	214	12995103	36.99	27.57065
chrX	108297368	108297812	445	108297679	73.98	37.68077

### Lentivirus-mediated PRMT3 downregulation leads to osteopenia phenotype in mice

**Fig. 5:**
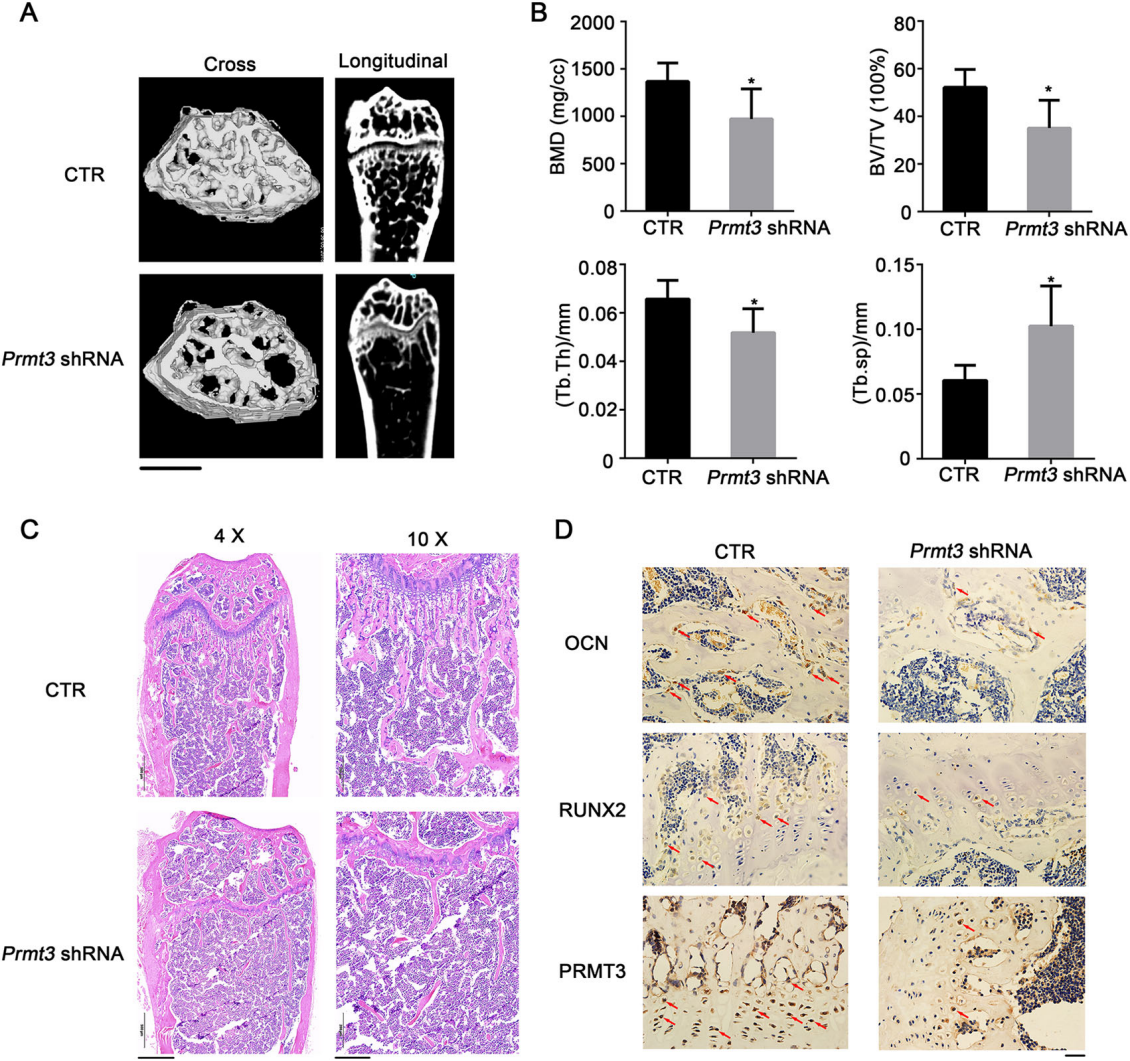
Lentivirus-mediated PRMT3 downregulation leads to osteopenia phenotype in mice. **a** Micro-CT image of the femurs from *Prmt3* shRNA treatment mice. Scale bar: 1 mm. **b** Quantitative measurements of bone mineral density (BMD), bone volume/tissue volume (BV/TV), trabecular thickness (Tb.Th), and trabecular spacing (Tb.Sp). Data are shown as mean ± SD; *n* = 5; *P < 0.05 by Student’s *t*-tests. **c** H&E staining of femur bone sections. Scale bar for 4×: 500 μm; Scale bar for 10×: 200 μm. **d** IHC staining for OCN, RUNX2, and PRMT3 of bone sections from *Prmt3* shRNA treatment mice. Scale bar: 20 μm. Red arrows indicate positive staining

Since PRMT3 plays a key role in the osteogenic differentiation of MSCs, we determined the effect of lentivirus-mediated PRMT3 downregulation on bone homeostasis in mice. *Prmt3* shRNA or negative control (NC) groups were injected through tail vein. Micro-CT analysis showed that *Prmt3* shRNA treatment reduced the number and thickness of trabecular bones (Fig. 5a). Bone histomorphometry analysis showed that the values of bone mineral density (BMD), bone volume/tissue volume (BV/TV), and trabecular thickness (Tb.Th) were reduced following *Prmt3* shRNA injection, and the trabecular spacing (Tb.Sp) was increased (Fig. 5b). To validate the micro-CT evaluation, histological analyses of bone tissue slices were performed. H&E staining showed that the trabecular bone volume of *Prmt3* shRNA mice was dramatically reduced in the distal femur compared with that in the NC group (Fig. 5c). The expression levels of PRMT3 and the osteogenesis markers RUNX2 and OCN were reduced in the compact bone and bone marrow in *Prmt3* shRNA mice, as indicated by immunohistochemical analyses (Fig. 5d). IHC staining of ALP also exhibited less positive marker in *Prmt3* shRNA mice (Supplementary Fig. 4a). Osteogenic differentiation and osteoclast differentiation are coupled in vivo. Tartrate-resistant acid phosphatase (TRAP) staining showed that treatment with *Prmt3* shRNA elevated the osteoclast number in mice (Supplementary Fig. 4b).

### The PRMT3 inhibitor SGC707 inhibits osteogenic differentiation of MSCs

**Fig. 6:**
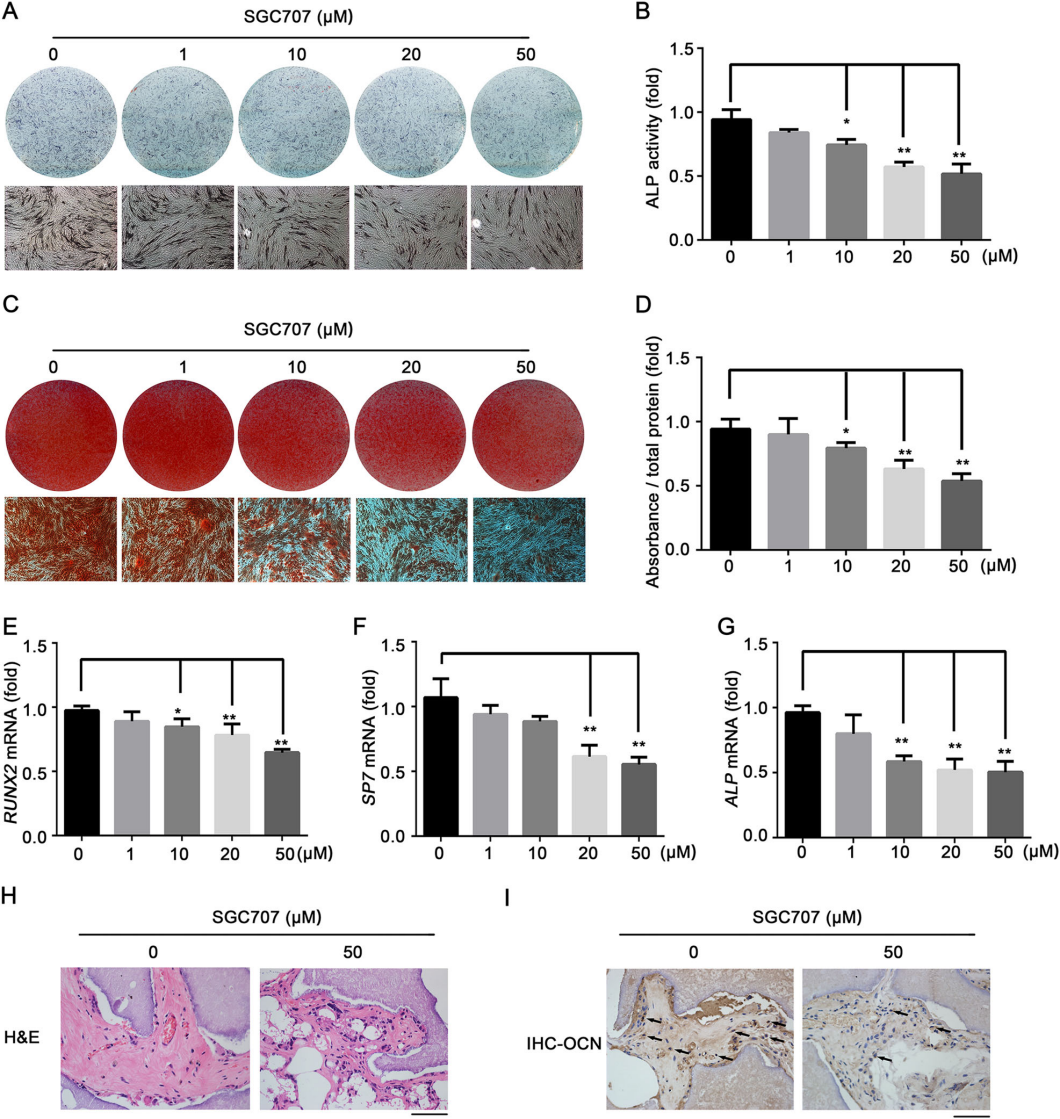
The PRMT3 inhibitor SGC707 inhibits osteogenic differentiation of MSCs. **a**, **b** ALP staining (**a**) and quantification (**b**) indicated that SGC707 treatment attenuated ALP activity in MSCs at 7 days after osteogenic induction. Data are shown as mean ± SD; *n* = 3; *P < 0.05 compared with vehicle (0 μM), **P < 0.01 compared with vehicle (0 μM) by ANOVA with Tukey’s post hoc test. **c**, **d** Alizarin Red Staining (**c**) and quantification (**d**) of MSCs following SGC707 treatment at 14 days after osteogenic induction. Data are shown as mean ± SD; *n* = 3; *P < 0.05 compared with vehicle (0 μM); **P < 0.01 compared with vehicle (0 μM) by ANOVA with Tukey’s post hoc test. **e–g** qRT-PCR analysis of mRNA expression levels of the osteogenesis-related genes *RUNX2* (**e**), *SP7* (**f**), and *ALP* (**g**) in MSCs at 7 days after osteogenic induction. Data are shown as mean ± SD; *n* = 3; *P < 0.05 compared with vehicle (0 μM); **P < 0.01 compared with vehicle (0 μM) by ANOVA with Tukey’s post hoc test. **h**, **i** The in vivo bone formation experiment. H&E staining (**h**) and IHC staining (**i**) for OCN of groups with or without treatment of SGC707. Scale bar: 50 μm. Black arrows indicate positive staining of OCN

To confirm our findings, we explored the effect of the selective PRMT3 inhibitor SGC707 on the osteogenic differentiation of MSCs. SGC707 binds to the allosteric site of PRMT3 and inhibits its methyltransferase activity in cells^35^. CCK8 assay showed that up to 50 μM SGC707 had no significant effect on the proliferation ability of MSCs (Supplementary Fig. 5a). Moreover, SGC707 significantly inhibited the expression of H4R3me2a at concentrations >10 μM (Supplementary Fig. 5b). MSCs were cultured in osteogenic medium following treatment with various concentrations of SGC707. ALP staining and quantification indicated that SGC707 treatment attenuated ALP activity in MSCs (Fig. 6a, b). Similarly, ARS staining and quantification indicated reduced calcium deposition in cells treated with SGC707 (Fig. 6c, d). Furthermore, SGC707 treatment downregulated the mRNA expression levels of the osteogenesis-related genes *RUNX2*, *SP7*, and *ALP* (Fig. 6e–g). We then confirmed the effect of SGC707 on osteogenesis by in vivo study. MSCs pre-treated with SGC707 (50 μM) for 96 h were mixed with β-TCP scaffolds and implanted subcutaneously in nude mice. As shown in Fig. 6h, H&E staining indicated that SGC707 treatment reduced eosinophilic bone-like tissue formation, and IHC staining of OCN indicated lower positive expression in cells treated with SGC707 (Fig. 6i).

### SGC707 treatment induces bone loss in mice

**Fig. 7:**
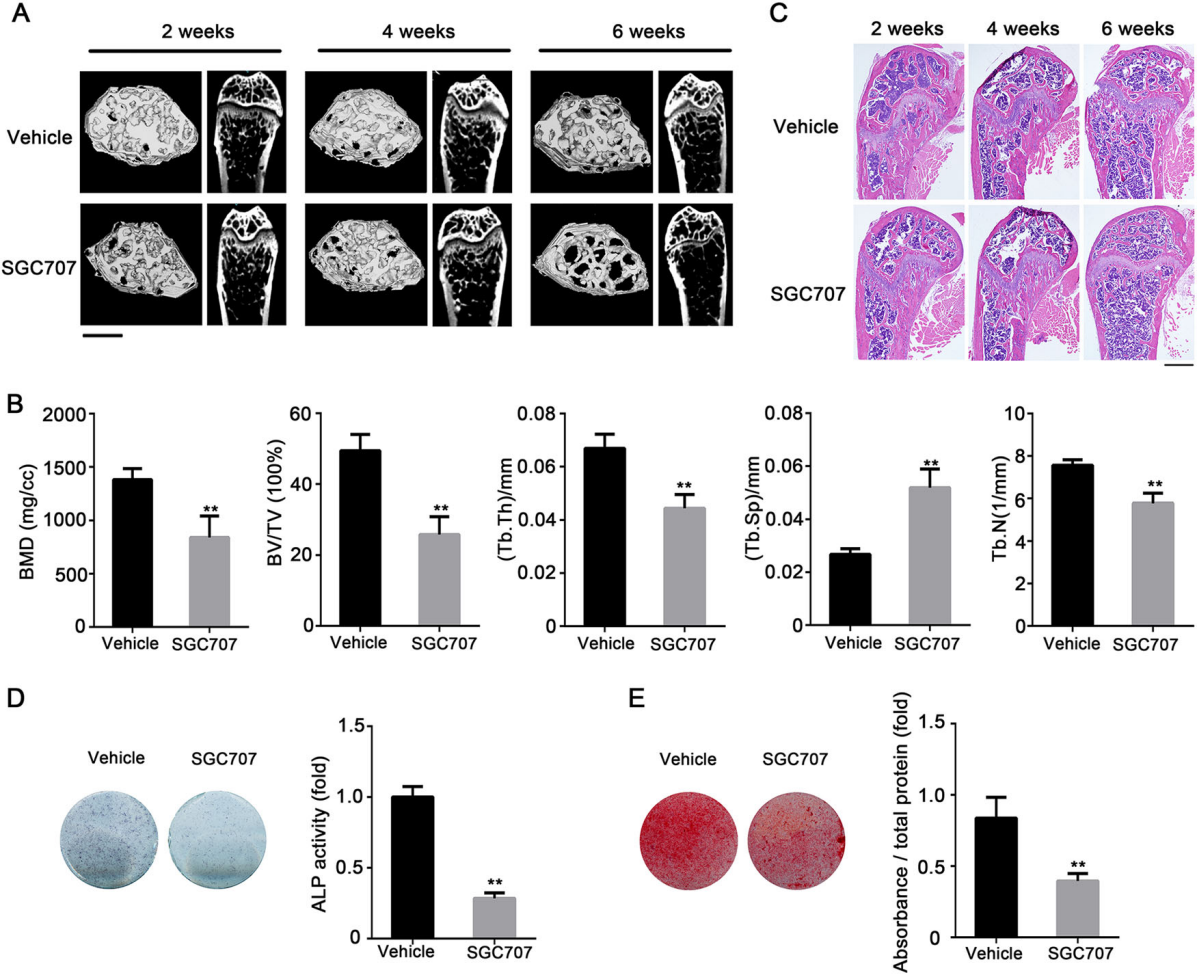
SGC707 treatment induces bone loss in mice. **a** Micro-CT image of trabecular bone in femurs at 2, 4, and 6 weeks after administration of SGC707 or vehicle. Scale bar:1 mm. **b** Quantitative measurements of bone mineral density (BMD), bone volume/tissue volume (BV/TV), trabecular number (Tb.N), trabecular thickness (Tb.Th), and trabecular spacing (Tb.Sp) of femurs with SGC707 delivery for 6 weeks. Data are shown as mean ± SD; *n* = 5; **P < 0.01 by Student’s *t*-tests. **c** H&E staining of trabecular bone in femurs at 2, 4, 6 weeks after SGC707 delivery. Scale bar: 500 μm. **d** ALP staining (left) and quantification (right) of mBMMSCs with SGC707 delivery or vehicle on day 7 after osteogenic induction. Data are shown as mean ± SD; *n* = 3; **P < 0.01 by Student’s *t*-tests. **e** Alizarin Red S Staining (left) and quantification (right) of mBMMSCs following SGC707 or vehicle treatment on day 14 after osteogenic induction. Data are shown as mean ± SD; *n* = 3; **P < 0.01 by Student’s *t*-tests

We determined whether SGC707 has an effect on bone homeostasis similar to that of intravenous injection of *Prmt3* shRNA. ALZET Pump Models were used for the controlled release of SGC707. Micro-CT analysis showed that the trabecular bone in femurs was decreased at 6 weeks with SGC707 delivery, whereas there was no significant difference between the vehicle and SGC707 groups at 2 and 4 weeks (Fig. 7a). Bone histomorphometry analysis showed that the values of BMD, BV/TV, trabecular number (Tb.N), and Tb.Th were reduced, and the trabecular spacing (Tb.Sp) was increased in mice treated with SGC707 for 6 weeks (Fig. 7b). There were no significant differences between the vehicle and SGC707 group at 2 and 4 weeks (Supplementary Fig. 6a–b). Moreover, H&E staining showed similar results to micro-CT analysis. The trabecular bone content and thickness of SGC707-treated mice were dramatically reduced in the distal femur compared with vehicle group at 6 weeks (Fig. 7c). We then evaluated the osteogenic capability of BMMSCs from mice treated with SGC707 for 6 weeks. Compared with the vehicle group, BMMSCs from SGC707-treated mice showed impaired ALP activity and less extracellular matrix mineralization (Fig. 7d, e). TRAP staining showed that treatment with SGC707 elevated the osteoclast number in mice (Supplementary Fig. 7).

## Discussion

Disruption of the balance between osteoblast and osteoclast function plays a vital part in the pathogenesis of osteoporosis^36,37^. OVX mice are typically used as an osteoporotic model^38^ to induce bone loss. Our study showed that BMMSCs separated from OVX mice exhibited lower ALP activity and mineralization. Moreover, PRMT3 expression level was increased during MSCs osteoblastic differentiation and decreased in BMMSCs from OVX mice, suggesting that PRMT3 plays a critical role in MSC-mediated osteogenesis and therefore the pathogenesis of osteoporosis. By using gain- and loss-of-function assays, we confirmed that PRMT3 enhances MSC osteoblastic differentiation in vitro and in vivo.

Epigenetic modification is an intrinsic modulatory mechanism in cell development and differentiation^39^. Imbalance of epigenetic regulation leads to various pathologies, including cancer, inflammatory diseases, and metabolic disorders^40–42^. Histone methylation is an important form of epigenetic regulation, and is associated with chromatin structure and regulation of gene expression. Histone methylation mainly occurs on lysine and arginine residues^43,44^. Lysine methylation is required for the subtle control of MSC differentiation and osteogenesis^45–47^. However, the involvement of histone arginine methylation in the lineage commitment of MSCs remains unclear. PRMT3 catalyzes asymmetric dimethylation of H4R3^23,48,49^. We confirmed that H4R3 is a substrate of PRMT3 in MSCs. Knockdown of PRMT3 decreased the H4R3me2a level in MSCs. The expression levels of H4R3me2a in BMMSCs from OVX mice or MSCs under osteogenic conditions were consistent with those of PRMT3. Most importantly, forced expression of catalytically mutant PRMT3 failed to rescue the osteogenic capacity of PRMT3-deficient MSCs. Thus, the asymmetric dimethylation of H4R3 catalyzed by PRMT3 is likely a novel regulatory mechanism underlying the osteogenic differentiation of MSCs.

MicroRNAs are single-stranded RNAs of about 22 nucleotides that induce post-transcriptional repression by binding to complementary sequences and leading to mRNA degradation or repression of translation^50^. Substantial research has been performed to reveal the function and targets of individual miRNAs such as miR-21, miR-375, and miR-138, which are associated with osteogenic differentiation of MSCs^51–53^. Transcriptional or epigenetic factors are key regulators of microRNA expression. For instance, PRMT4 contributes to the expression of several myogenic-related microRNA via dimethylation of H3R17 at upstream regulatory regions^54^. PRMT7 epigenetically repressed the expression of miR-24-2 by upregulating H4R3me2s level in the promoter region^55^. The H4R3me2a marker is a modification site associated with chromatin remodeling for activation of genes^56,57^, including pS2, IL17, and the β-globin locus^58–60^. Our results demonstrated that PRMT3 epigenetically increased the expression of miR-3648 by upregulating H4R3me2a. miR-3648 increases cell proliferation by direct downregulation of APC2^61^. However, the effect of miR-3648 on osteogenesis was unclear. Our results showed that overexpression of miR-3648 promoted osteogenic differentiation, while inhibition of miR-3648 repressed osteogenic differentiation in MSCs. Moreover, forced expression of miR-3648 could rescue the osteogenic capacity of PRMT3 knockdown cells. Our results indicate that miR-3648 is a novel regulator of MSC-mediated osteogenesis. Further investigation is required to verify the direct downstream osteogenesis-related targets of miR-3648 in MSCs.

SGC707 is a first-in-class PRMT3 chemical probe that binds to the allosteric site of PRMT3 and inhibits its methyltransferase activity in a temporal, selective, and dose-dependent manner^35^. We confirmed that SGC707 could inhibit PRMT3 catalytic activity and reduce the H4R3 asymmetric dimethylation level in MSCs, with no effect on the proliferation of cells. Moreover, SGC707 suppressed the osteogenic capacity of MSCs in a dose-dependent manner, which confirmed that PRMT3 promotes osteogenic differentiation via its methyltransferase activity.

Bone homeostasis is a complex and tightly regulated process, involving the balance between new bone formation carried out by MSC-derived osteoblasts and bone resorption achieved by hematopoietic stem cell-derived osteoclasts. Factors that regulate cell proliferation and cell fate decision of both cell types may lead to osteopenia and osteoporosis^62^. By systematic application of *Prmt3* shRNA and the specific inhibitor SGC707, we found that PRMT3 deficiency leads to bone loss in mice. The expression levels of RUNX2, OCN and ALP were reduced in bone sections of *Prmt3* shRNA mice. BMMSCs isolated from mice treated with SGC707 for 6 weeks showed decreased osteogenic capacity. Thus, the osteopenia phenotype could be attributed to a formation-resorption imbalance, wherein diminished bone formation could not compensate for regular or excessive bone resorption. PRMTs were recently reported to participate in the RANKL-induced osteoclastogenesis^20,21^. TRAP staining showed that treatment with *Prmt3* shRNA or SGC707 elevated the osteoclast number in mice. Further studies are needed to explore the detailed mechanism of how PRMT3 affects the function of osteoclasts and thus regulates the process of bone resorption. Joya et al. found that SGC707 treatment impairs the hepatic lipogenesis due to administration of LXR agonists as therapeutic agents for cholesterol-driven diseases in an in vivo animal study^49^. Our study revealed the effect of SGC707 on bone metabolism, which should be taken into consideration for clinical application.

In conclusion, our study revealed that PRMT3 plays an essential role in the osteoblastic differentiation of MSCs by epigenetically controlling miR-3648 transcription. *Prmt3* shRNA and SGC707 treatment resulted in bone reduction in mice. Our results suggested that PRMT3 is a novel regulator of bone homeostasis, and indicated that PRMT3 and H4R3me2a could be biomarkers and therapeutic targets for the diagnosis and treatment of osteoporotic diseases.

## Materials and methods

### Cell culture and lineage differentiation

Primary hMSCs were purchased from ScienCell Research Laboratories (Carlsbad, CA, USA). Cells at passage 3 from three independent donors were used for the assays. Proliferation media (DMEM; 10% fetal bovine serum; 1% penicillin/streptomycin) and osteogenic media [Proliferation media containing 100 nM dexamethasone (Sigma-Aldrich, St. Louis, MO, USA), 0.2 mM ascorbic acid (Sigma-Aldrich), and 10 mM β-glycerophosphate (Sigma-Aldrich)] were used for cell culture and osteogenic induction respectively.

### Isolation of primary mBMMSCs

The bone marrow was flashed out of mouse femurs and harvested. After centrifugation for 5 min at 1000 rpm, the cells were collected and cultured in fresh α-MEM mixed with 20% FBS and 1% antibiotics. Non-adherent cells were discarded at 48 h and attached cells were maintained until they reached full confluency. Mouse bone marrow mesenchymal stem cells (mBMMSCs) at passage 1 were used to analyze the expression levels of PRMT3 and H4R3me2a under osteoporotic conditions. Osteogenic media [fresh α-MEM containing 100 nM dexamethasone (Sigma-Aldrich, St. Louis, MO, USA), 0.2 mM ascorbic acid (Sigma-Aldrich), and 10 mM β-glycerophosphate (Sigma-Aldrich)] was used for osteoinduction of mBMMSCs.

### Alkaline phosphatase staining and quantification

Cells were cultured in 12-well plates and harvested after 7-day osteoinduction. The BCIP/NBT staining kit (CWBIO, Beijing, China) was used to for Alkaline phosphatase (ALP) staining. To determine the ALP activity, cells were rinsed three times with PBS and lysed with 1% TritonX-100 (Sigma-Aldrich) for 10 min on ice. The cells were then harvested and centrifuged at 12000 rpm for 30 min at 4 °C. BCA protein assay kits (Prod#23225, Pierce Thermo Scientific, Waltham, MA, USA) were used to detect protein concentration. ALP activity was measured using an ALP assay kit (A059-2, Nanjing Jiancheng Bioengineering Institute, Nanjing, China). The comparison of relative ALP activity among groups was quantitatively performed after normalization to the total protein concentration.

### Alizarin Red S staining and quantification

After osteogenic induction for 2 weeks, cells were fixed with 95% ethanol for 30 min at room temperature. After washing three times with distilled water, the cells were incubated with Alizarin Red S solution (2%, pH 4.2, Sigma-Aldrich). For the quantitative detection of calcium deposition, the staining samples were incubated with 100 mM cetylpyridinium chloride (Sigma-Aldrich) for 1 h and the absorbance was measured at 562 nm and normalized to the total protein concentration.

### RNA extraction, reverse transcription, and quantitative real-time PCR

Total RNA was extracted with TRIzol reagent (Invitrogen, Carlsbad, CA, USA). Reverse transcription was performed with a PrimeScript RT Reagent Kit (Takara, Tokyo, Japan). Real-time quantitative PCR assays were performed using Power SYBR Green PCR Master Mix and an ABI PRISM 7500 sequence detection system (Applied Biosystems, CA, USA). Glyceraldehyde-3-phosphate dehydrogenase (GAPDH) and U6 were used as internal standards for mRNA and microRNA, respectively. The primer sequences used in this study are shown in Table 2. Data were analyzed based on the 2^−ΔΔCt^ method.

**Table 2:** Sequences primers for real-time PCR or ChIP-qPCR

Genes	Forward primer	Reverse primer
Primers for real-time PCR		
* GAPDH*	TGTTCGACAGTCAGCCGCAT	CGCCCAATACGACCAAATCCGT
* RUNX2*	ACCACAAGTGCGGTGCAAAC	ACTGCTTGCAGCCTTAAATGACTCT
* ALP*	TAAGGACATCGCCTACCAGCTC	TCTTCCAGGTGTCAACGAGGT
* OCN*	CACCATGAGAGCCCTCACACTC	CCTGCTTGGACACAAAGGCTGC
* PRMT3*	GGACATCCATGTGCACGGCAA	AGTGTGTTTTGGTGCTCTGAGGG
* COL1A1*	CTGGCGCAGATGGTGTTGCT	TTCCAGTCAGACCCTTGGCAC
* U6*	CTCGCTTCGGCAGCACA	AACGCTTCACGAATTTGCGT
* miR-3648*	GCGAGCACAGAATTAATACGAC	AGCCGCGGGGATCGCCGAGGG
Primers for ChIP-qPCR		
Primer-1	GTGGTCTCTCGTCTTCTCCC	GTCGCACGAACGCCTGTC
Primer-2	GCCGATCCTCTTCTTCCCC	GGGAGAAGACGAGAGACCAC

### Western blotting

For total protein, cells were lysed in radioimmunoprecipitation assay (RIPA) buffer containing protease inhibitor mixture (Roche Applied Science, Mannheim, Germany). The lysates were harvested and centrifuged for 30 min at 12000 rpm and 4 °C. The Pierce BCA protein assay kit (Thermo Scientific) was utilized to detect the protein concentrations of the supernatants. Histone protein was extracted using the EpiQuik Total Histone Extraction Kit according to the manufacturer’s protocol (Epigentek, USA). Aliquots (20 μg) of the protein extracts were separated by 10% SDS PAGE and transferred to polyvinylidene difluoride membrane (Merck Millipore, Darmstadt, Germany). The membranes were blocked with 5% skim milk for 1 h and incubated with primary antibodies at 4 °C overnight. The ECL kit (CWBIO) was used to detect immunoreactive protein bands after incubation for 1 h with peroxidase-linked secondary antibodies at room temperature. The antibodies used were: anti-GAPDH, anti-PRMT3, anti-OCN (Abcam, Cambridge, UK), anti-H4, anti-RUNX2, anti-Flag (Cell Signaling, Danvers, MA, USA), and anti-H4R3me2a (Active Motif, Carlsbad, CA).

### Bone formation in vivo

Beta-tricalcium phosphate (β-TCP) (Bicon, Boston, MA, USA) scaffolds were utilized for the in vivo study. Cells were harvested when they reached 100% confluence, resuspended, and mixed with β-TCP for 1 h. The mixtures were then implanted into the dorsal surface of nude mice subcutaneously. Each mouse held two implantation sites, which contained six groups of cells: hMSCs/NC, hMSCs/PRMT3-sh1, hMSCs/PRMT3-sh2, hMSCs/Vector/PRMT3sh, hMSCs/WT-PRMT3/PRMT3sh, and hMSCs/Mut-PRMT3/PRMT3sh as described previously. The samples were harvested after 6-week implantation, fixed using 4% paraformaldehyde, and decalcified for 10 days in 10% EDTA (pH 7.4). The specimens were dehydrated and embedded in paraffin. Paraffin sections with 5-mm thickness were stained with haematoxylin and eosin (H&E) and Masson’s trichrome stain. Osteogenic differentiation was validated by immunohistochemical (IHC) staining for Osteocalcin (OCN) (anti-OCN, Abcam, Cambridge, UK). All animal studies were performed under the approval of Experimental Animal Ethic Branch, Peking University Biomedical Ethic Committee.

### Virus infection and plasmid constructions

Human and mice PRMT3 shRNA and the scramble negative control were purchased from GenePharma company (Suzhou, China). The shRNA target sequences for human PRMT3 were: *PRMT3*sh#1, 5ʹ-CAGCCTTGTAGCAGTGAGTGA-3ʹ and *PRMT3*sh#2, 5ʹ-CCTTGGGAGAAAGAAGAGTAT-3ʹ; those for mouse Prmt3 were: *Prmt3*sh#1, 5ʹ-CCTCATTGTGACCCTGACTTT-3ʹ and *Prmt3*sh#2, 5ʹ-CCTACGGTTGAATATATGAAT-3ʹ. For gene overexpression, full length PRMT3 and catalytic mutant PRMT3 were cloned into the pLNB vector with a mutant CBA promoter. The pLNB vector was resistant to blasticidin. Viral packaging was conducted as described previously^38,63^. Briefly, the pLNB vectors psPAX2 (Addgene) and pVSV-G (Clontech, USA) were co-transfected into HEK293T cells by using the PolyJet™ Transfection Reagent (SignaGen Laboratories, Rockville, MD, USA) to produce lentiviruses. The supernatant was collected 36, 48, and 60 h after transfection, filtered, and centrifuged with PEG-it™ (System Biosciences, SBI, Mountain View, CA, USA). The concentrated particles were titrated, aliquoted, and stored at −80 °C until further use. For lentivirus transfection, hMSCs was exposed to diluted viral supernatant with polybrene (5 μg/ml) for 24 h. For construction of the PRMT3 knockdown cell line, puromycin was applied to choose the stably transfected cells after transfection for 72 h. For construction of PRMT3 rescue cell line, PRMTsh cells were transfected with the vector, Flag-PRMT3 wild-type (WT), and Flag-PRMT3 mutant (Mut). Puromycin and blasticidin were used to select the stably double-transfected cells after 72 h of transfection.

### ChIP and sequencing

Briefly, cells were cross-linked with 1% formaldehyde, resuspended in lysis buffer (1% SDS, 10 mM EDTA, and 50 mM Tris-HCl [pH 8.0]) on ice for 3 min, and fragmented by sonication in RIPA ChIP buffer (0.5 mM EGTA, 140 mM NaCl, 10 mM Tris-HCl, pH 7.5, 1% TritonX-100, 0.01% SDS, 1 mM EDTA and protease inhibitor). The samples were then incubated overnight with Protein A/G-beads bound with antibodies [PRMT3 (Abcam, Cambridge, UK), H4R3me2a (Active Motif, Carlsbad, CA), H3 (Cell Signaling)]. After the crosslinking was reversed, immune complexes containing DNA were purified and eluted. The precipitated DNA for sequencing, including H4R3me2a and PRMT3, were constructed into libraries with NEBNext DNA Library Prep Reagent Set for Illumina (NEB) as described by the manufacturer.

### ChIP-seq data analysis

Raw reads with adapters were trimmed and low-quality reads were discarded. We mapped the clean reads to a human reference genome (hg19) by using Burrows-Wheeler Aligner (BWA; ref1), retaining only unique non-duplicate reads. Fragment sizes were predicted by the following command: macs2 predictd -i unique_non_duplicated_PRMT3.bed -f BED -g 3.1e9 -outdir./. Peaks were called by MACS (ref2) using input as controls by the following command: macs2 callpeak -t unique_non_duplicated_PRMT3.bed -c $input.bed -g 3.1e9 -keep-dup all -nomodel -extsize $predicted_fragment_size -q 0.01.

### Transient transfection

Cells were seeded at 70–80% confluency and transfected with miR-3648 mimic, miR-3648 mimic control, miR-3648 inhibitor, or miR-3648 inhibitor control (Ribobio, Guangzhou, China) at 100 nM by using Lipofectamine 2000 (Invitrogen, Carlsbad, CA). The media was changed 6 h after transfection

### Cell proliferation assay

The cell proliferation assay was performed with the Cell Counting Kit-8 (Dojindo, Kumamoto, Japan) according to the manufacturer’s protocol. The media from each group was removed at the requisite time-point and cells were subsequently cultured in CCK-8-containing DMEM media for 2 h at 37 °C. Absorbance of each group was examined at 450 nm by using a microplate reader.

### Measurement of micro-computed tomography for bone samples

Eight-week-old female C57BL/6 mice were provided by Vital River Corporation (Beijing, China) and maintained in pathogen-free facilities with a 12-h light/dark cycle. After 1 week, ovariectomy (OVX) or sham operation was bilaterally conducted by standard methods under general anesthesia with pentobarbital sodium injection (50 mg/kg)^64^. The mice were sacrificed by CO_2_ asphyxiation at 6 or 12 weeks. The femurs were harvested and analyzed by high-resolution microtomography (Inveon, Siemens, Munich, Germany). Images were obtained at an effective pixel size of 8.82 μm, voltage of 80 kV, tube current of 500 μA, and exposure time of 1500 ms in each of the 360 rotational steps. Parameters were analyzed by Inveon Research Workplace (Siemens), and included BV/TV, Tb.Th, and Tb.N according to the guidelines set by the American Society for Bone and Mineral Research (ASBMR).

### Mini-osmotic pump (ALZET) implantation

Mice were randomly assigned to six groups of five animals each. The groups were (1) 2 week/CTR, (2) 2 week/SGC707, (3) 4 week/CTR, (4) 4 week/SGC707, (5) 6 week/CTR, and (6) 6 week/SGC707. After the mice were anesthetized with pentobarbital sodium injection (50 mg/kg), the backs were subcutaneously implanted with an Alzet mini-pump (model 2002, Alzet, Cupertino, CA, USA). The Alzet pumps were used with a delivery rate at 0.5 μL/h, or 200 μL over 2 weeks. To ensure continuous exposure during the 4- or 6-week period, two or three mini-osmotic pumps were consecutively implanted. Alzet pumps were filled according to the manufacturer’s instructions. To achieve a mean targeted concentration of 20 mg/kg/day, SGC707 was dissolved in DMSO at a concentration of 28 mg/ml. After ALZET implantation for 2, 4, or 6 weeks, the mice were euthanized and the femurs were harvested. The femurs were fixed in 4% paraformaldehyde for 24 h and scanned by micro-CT. Then the femurs were embedded in paraffin and sectioned to conduct H&E staining and IHC staining for PRMT3 (Abcam, Cambridge, UK), RUNX2 (Cell Signaling, Danvers, MA, USA), and OCN (Abcam, Cambridge, UK).

### Statistical analyses

The statistical analyses were done with SPSS Statistics 20.0 software (IBM, Armonk, NY, USA). Comparisons between two groups were conducted by Student’s *t*-tests, and multiple comparisons were performed by one-way analysis of variance (ANOVA) followed by a Tukey’s post hoc test. Data were shown as the mean ± standard deviation (SD) of three assays per group. *P*-values < 0.05 were considered statistically significant.

### Accession number

All of the ChIP-seq data were deposited in GEO under the accession number GSE124674. To review GEO accession GSE124674: Go to Enter token adurqkkihfcfbkb into the box.

## Supplementary information

Supplementary figure legends

Supplementary figure 1

Supplementary figure 2

Supplementary figure 3

Supplementary figure 4

Supplementary figure 5

Supplementary figure 6

Supplementary figure 7
