# Supplementary material for: Asymmetrical methyltransferase PRMT3 regulates human mesenchymal stem cell osteogenesis via miR-3648
Source: Cell Death Dis. 2019 Aug 5;10(8):581. doi: 10.1038/s41419-019-1815-7 (PMC6680051; doi:10.1038/s41419-019-1815-7)
Supplement: Supplementary file 1 — Supplementary figure legends [file 41419_2019_1815_MOESM1_ESM.docx]

**Supplementary figure legends**

**Supplementary Figure 1.**

A Quantitative measurement of bone mineral density (BMD), bone volume/tissue volume (BV/TV), trabecular thickness (Tb.Th), and trabecular spacing (Tb.Sp) in OVX mice at 6 weeks. Data are shown as mean ± SD; n = 6; *: *P* < 0.05, **: *P* < 0.01 by Student’s *t*-tests.

B Quantitative measurement of bone mineral density (BMD), bone volume/tissue volume (BV/TV), trabecular thickness (Tb.Th), and the trabecular spacing (Tb.Sp) in OVX mice at 12 weeks. Data are shown as mean ± SD; n = 6; **: *P* < 0.01 by Student’s *t*-tests.

C ALP staining (left) and quantification (right) of mBMMSCs in OVX mice at 6 weeks, 7 days after osteogenic induction. Data are shown as mean ± SD; n = 6; **: *P* < 0.01 by Student’s *t*-tests.

D ALP staining (left) and quantification (right) of mBMMSCs in OVX mice at 12 weeks, 7 days after osteogenic induction. Data are shown as mean ± SD; n = 6; **: *P* < 0.01 by Student’s *t*-tests.

**Supplementary Figure 2.**

A Transduction efficiency of PRMT3 lentivirus in hMSCs validated by fluorescence microscopy. Scale bar : 100 μm.

B Knockdown of PRMT3 was validated by qRT-PCR. Data are shown as mean ± SD; n = 6; **: *P* < 0.01 compared with NC by Student’s *t*-tests.

C qRT-PCR analysis of mRNA levels of PRMT3 in the NC, Vector/PRMT3sh, WT-PRMT3/PRMT3sh, and Mut-PRMT3/PRMT3sh groups. Data are shown as mean ± SD; n = 3; **: *P* < 0.01 compared with NC by Student’s *t*-tests.

**Supplementary Figure 3.**

A Overexpression efficiency of miR-3648 in hMSCs.

B Inhibition efficiency of miR-3648 in hMSCs.

C-D ALP staining (C) and quantification (D) in miR-3648 inhibition cells on day 7 after osteogenic induction. Data are shown as mean ± SD; n = 3; **: *P* < 0.01 by Student’s *t*-tests; miR-3648 inh., miR-3648 inhibitor.

E-F Alizarin Red staining (E) and quantification (F) in miR-3648 inhibition cells after osteogenic induction for 14 days. Data are shown as mean ± SD; n = 3; **: *P* < 0.01 by Student’s *t*-tests; miR-3648 inh., miR-3648 inhibitor.

**Supplementary Figure 4.**

A IHC staining for ALP of bone sections from Prmt3 shRNA treatment mice. Scale bar: 20 μm. Red arrows indicate positive staining.

B Tartrate-resistant acid phosphatase (TRAP) staining indicates osteoclasts at the distal metaphysis growth plate area of femurs from Prmt3 shRNA treatment mice. Scale bar, 20μm.

**Supplementary Figure 5.**

A CCK8 assay of hMSCs cultured with different concentrations of SGC707.

B SGC707 treatment inhibited the expression of H4R3me2a in hMSCs. hMSCs were treated with SGC707 for 48 h before collection.

**Supplementary Figure 6.**

A Quantitative measurements of bone mineral density (BMD), bone volume/tissue volume (BV/TV), trabecular thickness (Tb.Th), and trabecular spacing (Tb.Sp) of femurs with SGC707 delivery for 2 weeks. Data are shown as mean ± SD; n = 5

B Quantitative measurement of bone mineral density (BMD), bone volume/tissue volume (BV/TV), trabecular thickness (Tb.Th), and trabecular spacing (Tb.Sp) of femurs with SGC707 delivery for 4 weeks. Data are shown as mean ± SD; n = 5.

**Supplementary Figure 7.**

TRAP staining indicates osteoclasts at the distal metaphysis growth plate area of femurs of mice under SGC707 treatment for 6 weeks. Scale bar, 20μm.
