# Supplementary figures and images for: Asymmetrical methyltransferase PRMT3 regulates human mesenchymal stem cell osteogenesis via miR-3648
Source: Cell Death Dis. 2019 Aug 5;10(8):581. doi: 10.1038/s41419-019-1815-7 (PMC6680051; doi:10.1038/s41419-019-1815-7)

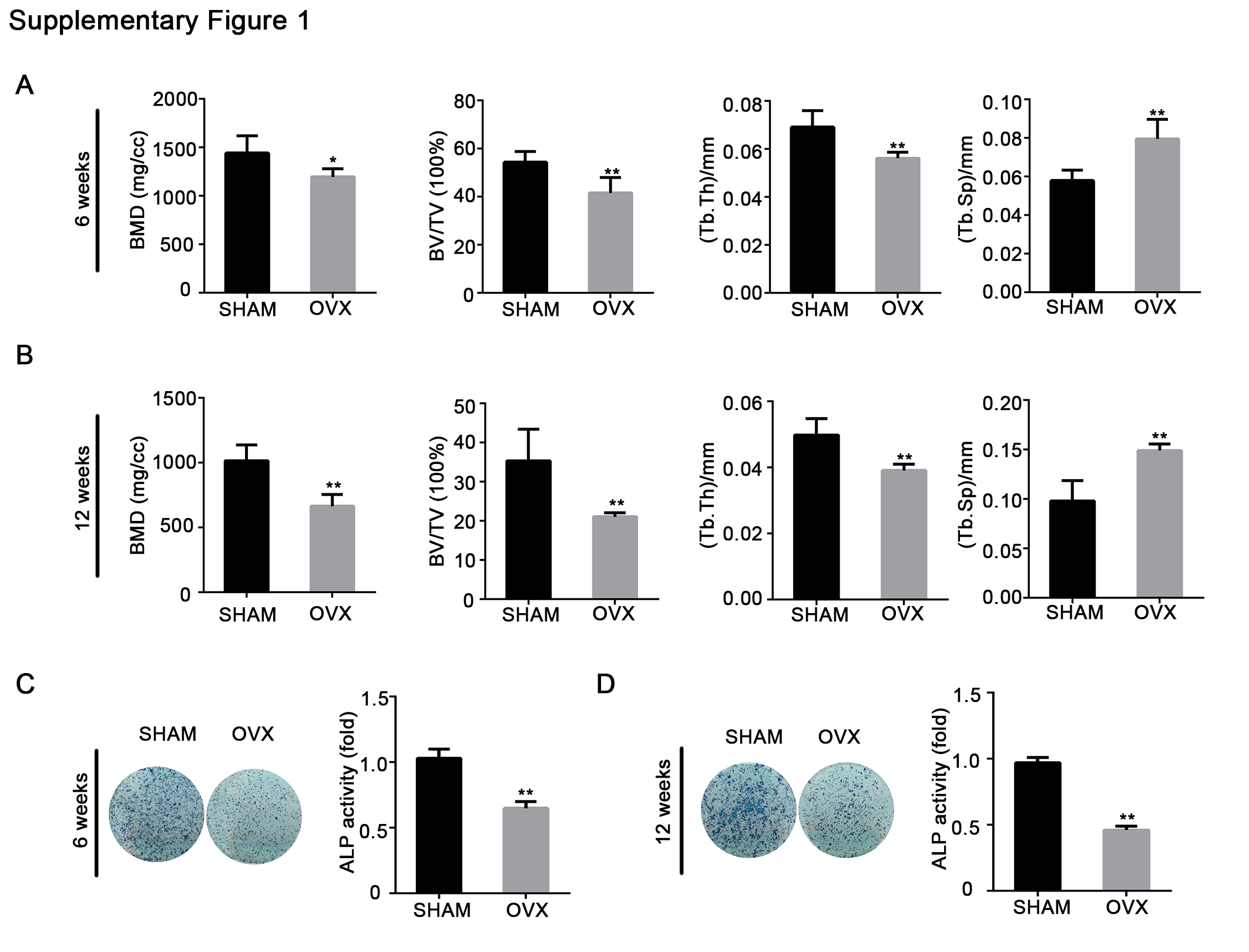

Supplement: Supplementary file 2 — Supplementary figure 1 [file 41419_2019_1815_MOESM2_ESM.tif]

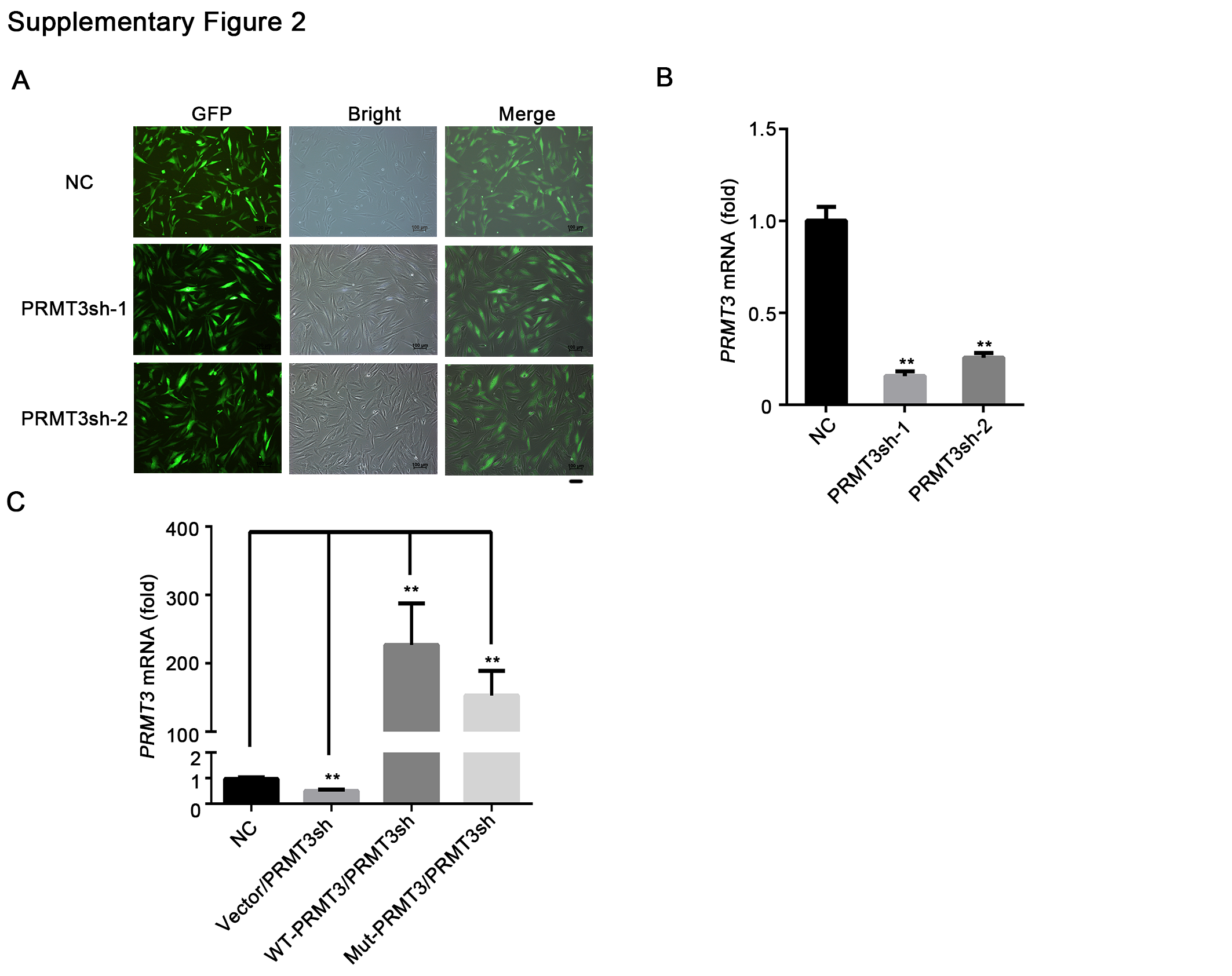

Supplement: Supplementary file 3 — Supplementary figure 2 [file 41419_2019_1815_MOESM3_ESM.tif]

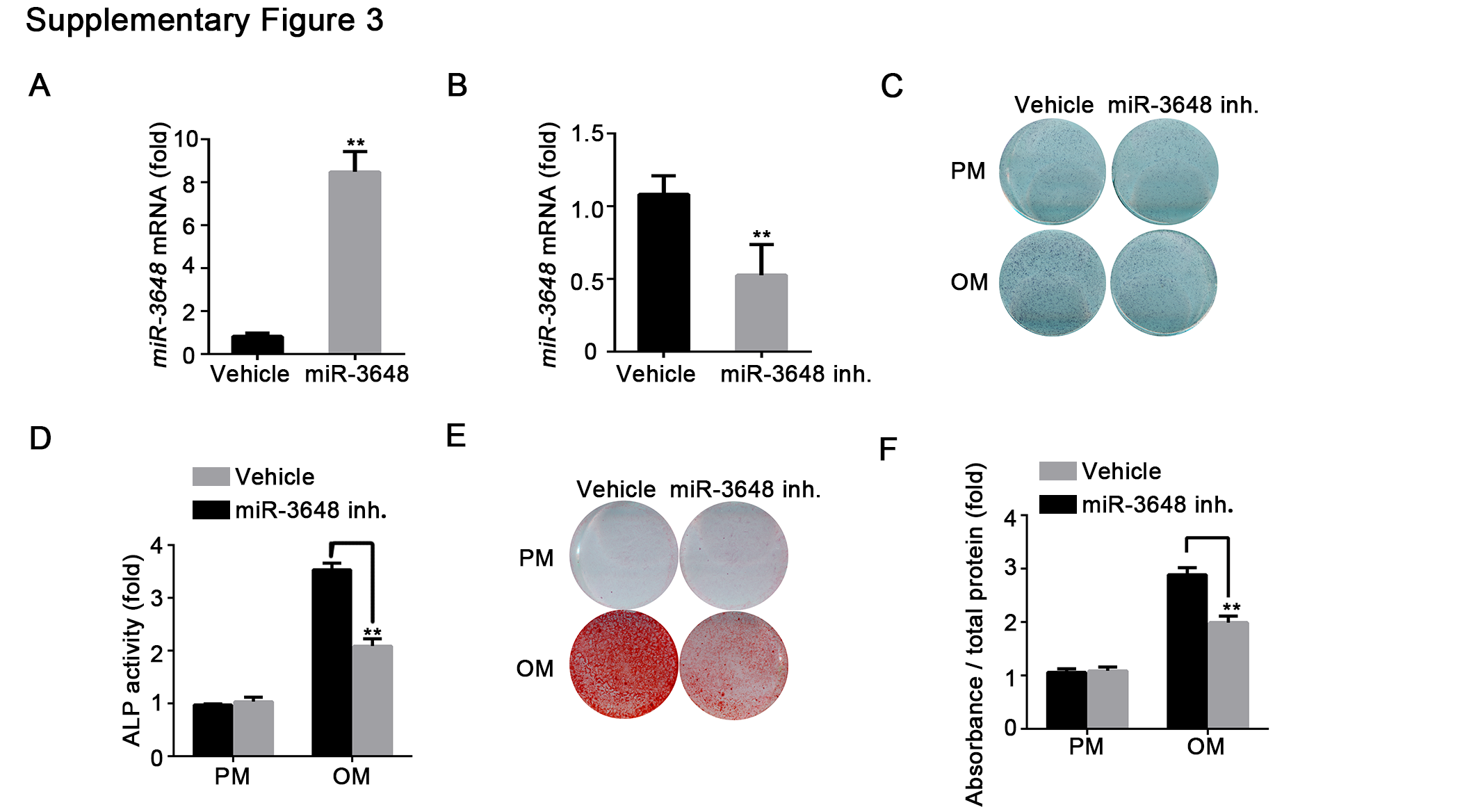

Supplement: Supplementary file 4 — Supplementary figure 3 [file 41419_2019_1815_MOESM4_ESM.tif]

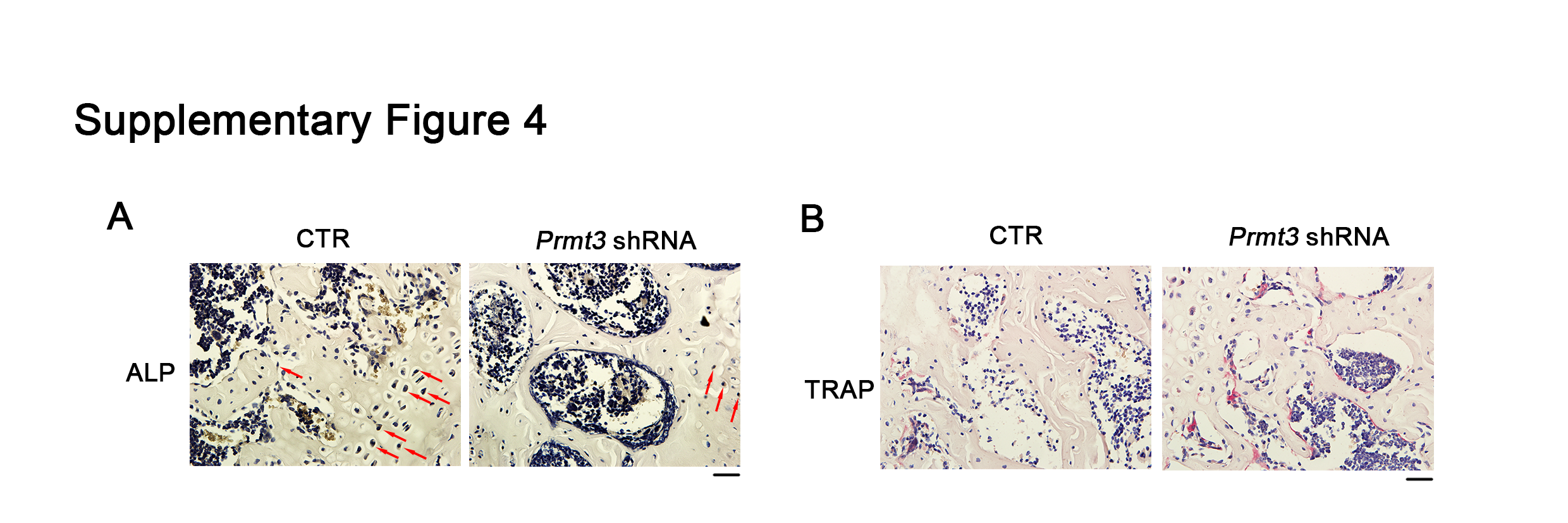

Supplement: Supplementary file 5 — Supplementary figure 4 [file 41419_2019_1815_MOESM5_ESM.tif]

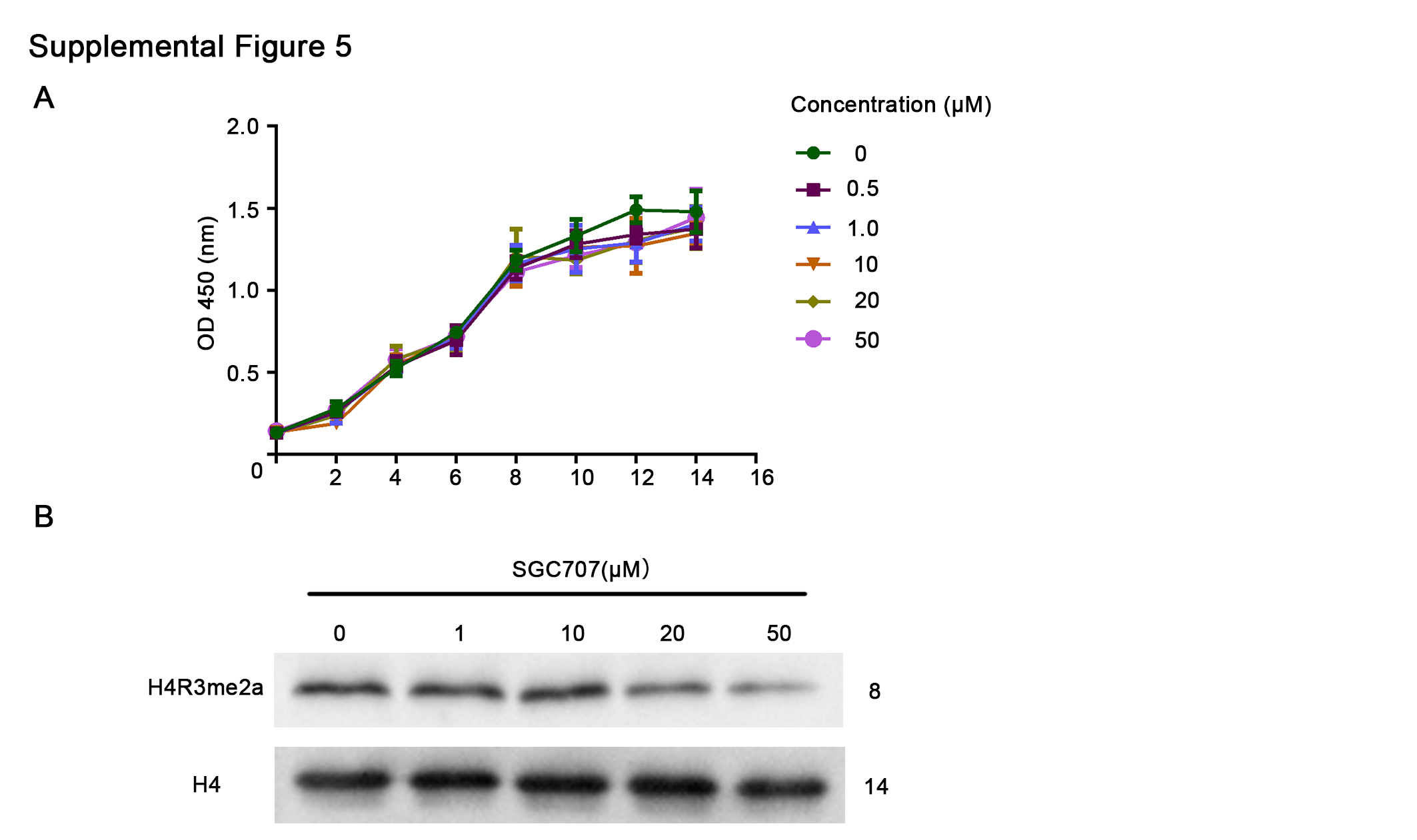

Supplement: Supplementary file 6 — Supplementary figure 5 [file 41419_2019_1815_MOESM6_ESM.tif]

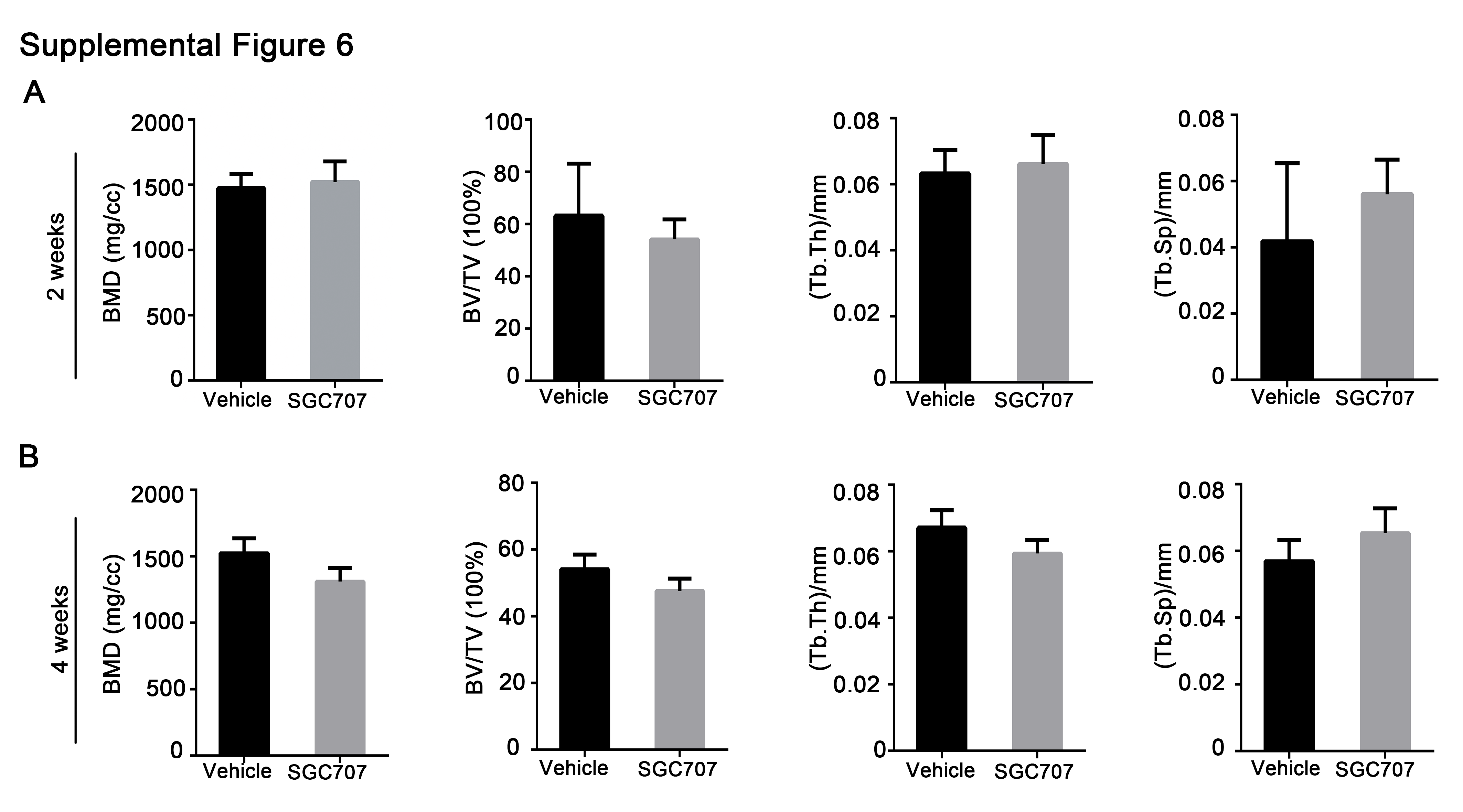

Supplement: Supplementary file 7 — Supplementary figure 6 [file 41419_2019_1815_MOESM7_ESM.tif]

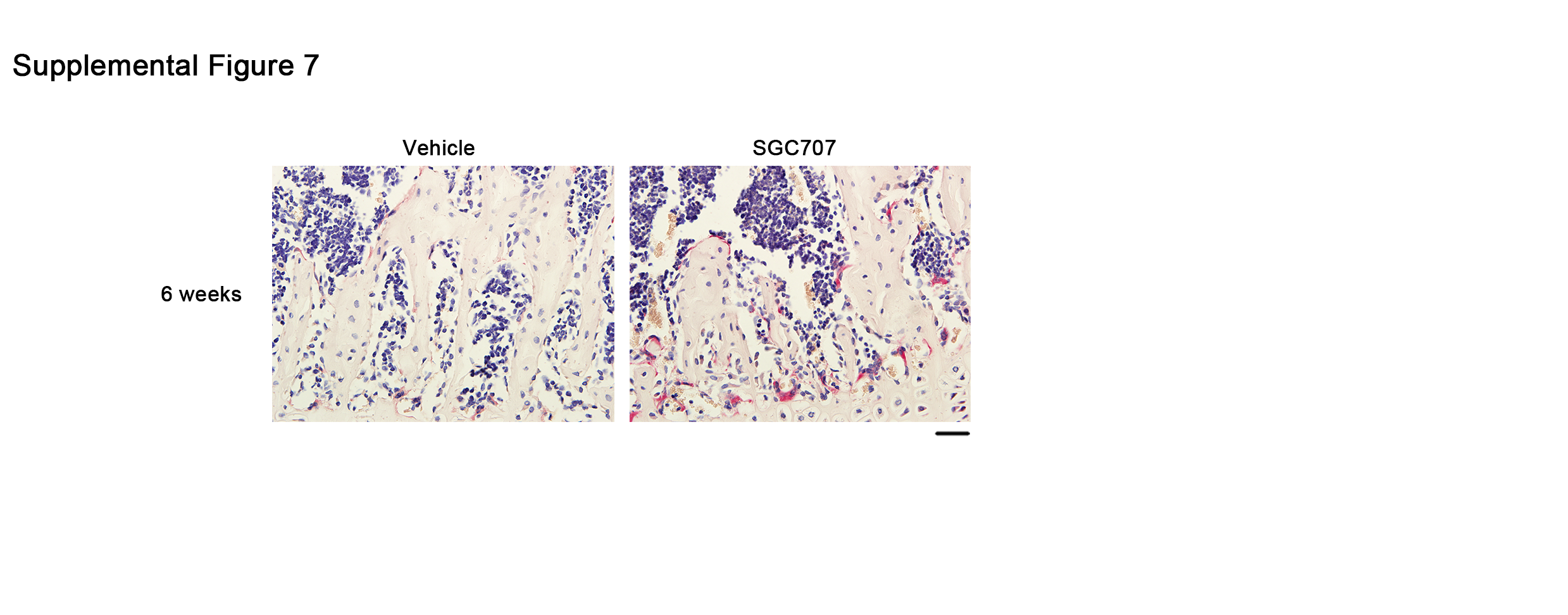

Supplement: Supplementary file 8 — Supplementary figure 7 [file 41419_2019_1815_MOESM8_ESM.tif]
